# Quantitative Brain MRI Metrics Distinguish Four Different ALS Phenotypes: A Machine Learning Based Study

**DOI:** 10.3390/diagnostics13091521

**Published:** 2023-04-24

**Authors:** Venkateswaran Rajagopalan, Krishna G. Chaitanya, Erik P. Pioro

**Affiliations:** 1 Department of Electrical and Electronics Engineering, Birla Institute of Technology and Science Pilani, Hyderabad Campus, Hyderabad 500078, India; 2 Neuromuscular Center, Department of Neurology, Neurological Institute, Cleveland Clinic, Cleveland, OH 44195, USA; 3 Department of Neurosciences, Lerner Research Institute, Cleveland Clinic, Cleveland, OH 44195, USA

**Keywords:** ALS phenotypes, MRI, machine learning, Random Forest, neural network

## Abstract

Amyotrophic lateral sclerosis (ALS) is a fatal neurodegenerative disease whose diagnosis depends on the presence of combined lower motor neuron (LMN) and upper motor neuron (UMN) degeneration. LMN degeneration assessment is aided by electromyography, whereas no equivalent exists to assess UMN dysfunction. Magnetic resonance imaging (MRI) is primarily used to exclude conditions that mimic ALS. We have identified four different clinical/radiological phenotypes of ALS patients. We hypothesize that these ALS phenotypes arise from distinct pathologic processes that result in unique MRI signatures. To our knowledge, no machine learning (ML)-based data analyses have been performed to stratify different ALS phenotypes using MRI measures. During routine clinical evaluation, we obtained T1-, T2-, PD-weighted, diffusion tensor (DT) brain MRI of 15 neurological controls and 91 ALS patients (UMN-predominant ALS with corticospinal tract CST) hyperintensity, *n* = 21; UMN-predominant ALS without CST hyperintensity, *n* = 26; classic ALS, *n* = 23; and ALS patients with frontotemporal dementia, *n* = 21). From these images, we obtained 101 white matter (WM) attributes (including DT measures, graph theory measures from DT and fractal dimension (FD) measures using T1-weighted), 10 grey matter (GM) attributes (including FD based measures from T1-weighted), and 10 non-imaging attributes (2 demographic and 8 clinical measures of ALS). We employed classification and regression tree, Random Forest (RF) and also artificial neural network for the classifications. RF algorithm provided the best accuracy (70–94%) in classifying four different phenotypes of ALS patients. WM metrics played a dominant role in classifying different phenotypes when compared to GM or clinical measures. Although WM measures from both right and left hemispheres need to be considered to identify ALS phenotypes, they appear to be differentially affected by the degenerative process. Longitudinal studies can confirm and extend our findings.

## 1. Introduction

Amyotrophic lateral sclerosis (ALS) is a fatal neurodegenerative disease whose diagnosis depends on the presence of degenerating motor neurons in both peripheral nervous system (PNS, or lower motor neuron (LMN)) and central nervous system (CNS, or upper motor neuron (UMN)) regions. Assessment of LMN degeneration is aided by widely used electromyography of peripheral nerve and muscle, while similar quantitative methods to assess CNS dysfunction are not easily accessible. Qualitative magnetic resonance imaging (MRI) is primarily used clinically to exclude conditions mimicking ALS. Although ALS patients eventually develop clinical evidence of both UMN and LMN dysfunction, some present with varying proportions of the two, and in a minority, demonstrate prominent frontotemporal cognitive impairment. Therefore, depending on the presence of such features, we have identified four phenotypes of ALS patients: (a) classic ALS (ALS-Cl) patients with obvious LMN and UMN clinical signs; (b) UMN-predominant ALS patients with prominent spasticity and few or no LMN signs at time of brain MRI, which reveals bilateral corticospinal tract (CST) hyperintensity on conventional FLAIR, T2-, and proton-density (PD)-weighted sequences (ALS-CST+) [1]; (c) UMN-predominant ALS patients with brain MRI showing no CST hyperintensity (ALS-CST−); and (d) ALS patients with frontotemporal dementia (ALS-FTD). We hypothesize that these different ALS phenotypes arise from distinct pathologic mechanisms that result in unique MRI signatures. 

In our recent studies [2,3,4,5], we used different quantitative MRI approaches to study brain degeneration in the above-mentioned ALS subgroups. These independent imaging approaches revealed distinct abnormalities in the gray matter (GM) and white matter (WM) brain regions of these phenotypes. Here, we aim to use machine learning (ML) algorithms to identify which clinical and MRI features are most important in accurately classifying the ALS subgroups. The features identified by ML can then be used to predict classification of newer data sets into one of the ALS phenotypes based on their clinical and MRI signatures. This will potentially allow stratification of ALS patients into specific phenotypes with different clinical progressions and potentially diverse disease mechanisms. 

ML algorithms have been employed in ALS to monitor disease progression (regression-based approach) [6,7], compare staging systems [8], and stratify patients based on clinical assessments such as limb involvement [9], disease stage, and ALSFRS-R score [10]. To date, ML models stratifying ALS patients have used category labels of ‘healthy’ and ‘ALS’ for all types, without separating into different phenotypes, as described above. The different ML methods used in ALS studies include support vector machine (SVM), discriminant analysis [11], Random Forest (RF) [8], canonical discriminant function [12], boosting [13], and artificial neural networks (ANN) [14]. In ALS studies, RF is the most commonly employed ML method and is one of the best-performing algorithms [15].

Because diagnosis of ALS is delayed an average of 12 months from symptom onset, survival is usually 2–4 years from symptom onset [16], there is an urgent need to identify non-invasive biomarkers for ALS, including those based on neuroimaging. At time of ALS diagnosis, WM degeneration is already observed with little subsequent deterioration. In contrast, GM degeneration progresses rapidly in the post-symptomatic phase [15]. Therefore, WM measures are believed to be more suitable for diagnostic models of ML, while changes in GM measures may reflect disease progression [15].

Important contributions of this study include identifying: (a) four ALS phenotypes using MRI, clinical, and demographic measures; (b) extensive and sophisticated feature sets that can detect changes at microscopic (diffusion measures) and macroscopic (graph measures such as global efficiency, fractal dimension (FD) analysis of brain WM/GM skeleton, general structure, etc.) levels; (c) feature sets of WM measures (specific to the universally affected CST and whole brain network), GM measures (simple brain parenchymal fraction [BPF] to sophisticated shape morphometric features such as FD) and bifurcation of clinical measures (such as the ALSFRS-R score and its constituent subscores); (d) different ML methods (by considering the complete vs selected feature sets) to stratify ALS phenotypes; (e) large clinical sample size (106 subjects) reflecting real-world data and a large feature set (121 features); and (f) which of WM, GM, demographic, and clinical measures play (a) dominant role(s) in diagnosing ALS patients. A future goal would be to understand how each feature selected by ML methods reflects the disease-related pathologic changes unique to each ALS phenotype. 

There is great interest in developing neuroimaging-based biomarkers of ALS to establish earlier diagnosis, recognize disease subtypes, monitor disease progression, and assess efficacy of therapeutic interventions. We aimed to identify the attributes (in our quantitative MRI measures) which may distinguish and diagnostically classify these four ALS phenotypes. In Methods, we present the MR analyses and ML algorithms used; in Results, we present findings from the ML algorithms and ML applied on different aspects of feature sets; and in Discussion, the findings are analyzed and their implications in each of ALS phenotypes.

## 2. Methods

### 2.1. Imaging Data

Patients with ALS and neurological controls underwent brain MRI on a 1.5T Siemens Symphony scanner (Erlangen, Germany) as part of routine clinical evaluation. High-resolution 3D T1-weighted axial images were obtained using magnetization-prepared rapid gradient echo (M-PRAGE) sequence with the following parameters: repetition time (TR) = 1800 ms, echo time (TE) = 4.38 ms, flip angle = 10°, inversion time (TI) = 1100 ms, slice thickness = 1 mm, in-plane resolution = 0.9 × 0.9 mm^2^, and number of slices = 160. T2- and PD-weighted images were used to identify WM pathology, including the presence of CST hyperintensity. T2- and PD- weighted images were acquired using a dual-echo fast spin-echo sequence with the following parameters: TR = 3900 ms, TE = 26 ms and 104 ms, echo train length or turbo factor = 7, number of averages = 1, slice thickness = 4 mm, and in-plane resolution = 0.9 × 0.9 mm. Diffusion tensor imaging (DTI) was performed using single-shot echo planar imaging (SS-EPI) in 12 diffusion-weighted directions with b = 1000 s/mm^2^ and a non-diffusion-weighted image with b = 0 s/mm^2^. Other parameters included: resolution = 1.9 × 1.9 × 4 mm^3^, TR = 6000 ms, TE = 121 ms, and EPI factor = 128.

Brain MRI of 91 ALS patients (ALS-CST+, *n =* 21; ALS-CST−, *n =* 26; ALS-Cl, *n =* 23; and ALS-FTD, *n =* 21) and 15 neurological controls were identified for storage, and analysis of deidentified images after patient verbal consent, as approved by the Cleveland Clinic Institutional Review Board.

### 2.2. Data Processing

**Figure 1:**
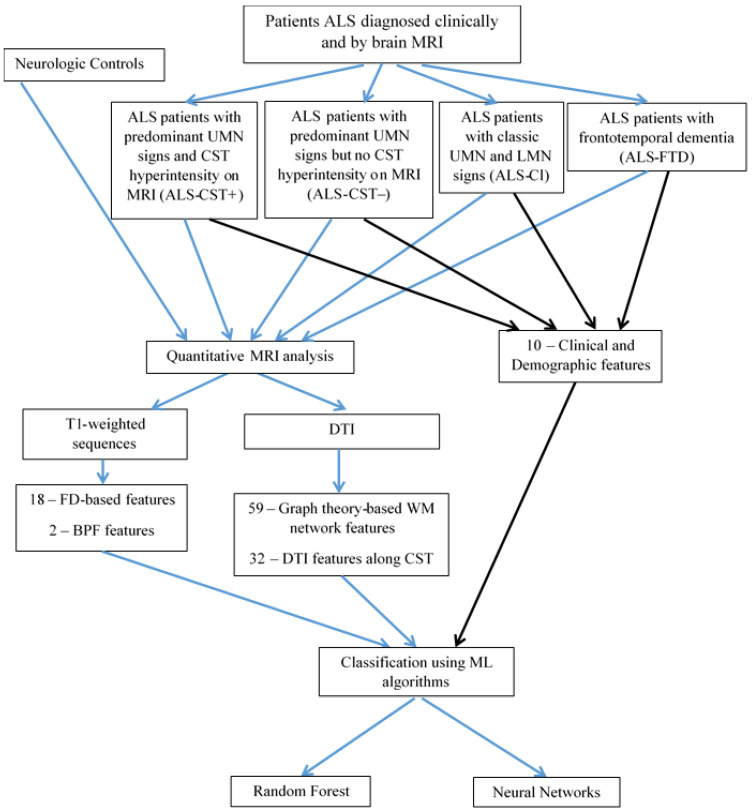
Diagram showing the workflow used in this study. ALS—Amyotrophic lateral sclerosis, BPF—Brain parenchymal fraction, CST—Corticospinal tract, DTI—Diffusion tensor imaging, FD—Fractal dimension, ML—Machine learning, WM—White matter.

We obtained 111 quantitative measures (attributes) from the MRI data and 10 non-imaging attributes (2 demographic and 8 clinical measures of ALS), as listed in Table 1. Although details of the image processing methods and quantitative metrics to obtain these attributes have been previously reported [17,18,19,20], they are briefly described below. A flowchart outlining our present study is shown in Figure 1.

### 2.3. Graph Network Features

Graph theory metrics were obtained from DTI data as follows: (a) preprocessing of DTI data using ExploreDTI (http://www.exploredti.com/ access data 10 January 2019) openware [21]; (b) fitting of diffusion data to the DTI model after correcting for motion artifact by using robust diffusion tensor estimation; (c) performance of brain tractography using deterministic streamline approach with a fractional anisotropy (FA) starting threshold of 0.2 and a stopping threshold of 1.0; (d) obtainment of connectivity matrices between cortical regions based on the AAL atlas [22], which were registered to the subject’s space; (e) construction of connectivity matrices for DTI metrics of FA, axial diffusivity (AD), radial diffusivity (RD) and mean diffusivity (MD); (f) performance of whole-brain WM network analysis using Graph Analysis toolbox (GAT) software (https://www.nitrc.org/projects/gat/ access data 10 January 2019) [23]. Microarchitectural tissues correlates of these DTI measures are believed to be: FA, both myelin and axonal integrity; RD, myelin integrity; AD, axonal integrity; and MD, mobility of water molecules.

The connectivity matrix obtained using ExploreDTI was used in GAT for further processing. We obtained network measures for each subject using the “network measures” module in GAT. Graph measures studied, as listed in Table 1, included: assortativity, density, mean clustering coefficient, transitivity, global efficiency, mean local efficiency, modularity, Louvian modularity, characteristic path length, mean nodal betweenness, mean edge betweenness, normalized path length, normalized clustering, small world index, and mean degree. Further details have been previously published [2,24]. DTI-derived WM measures from graph theory network analyses are shown in Table 1, lines 1–59.

### 2.4. DTI Corticospinal Tract Features

**Figure 2:**
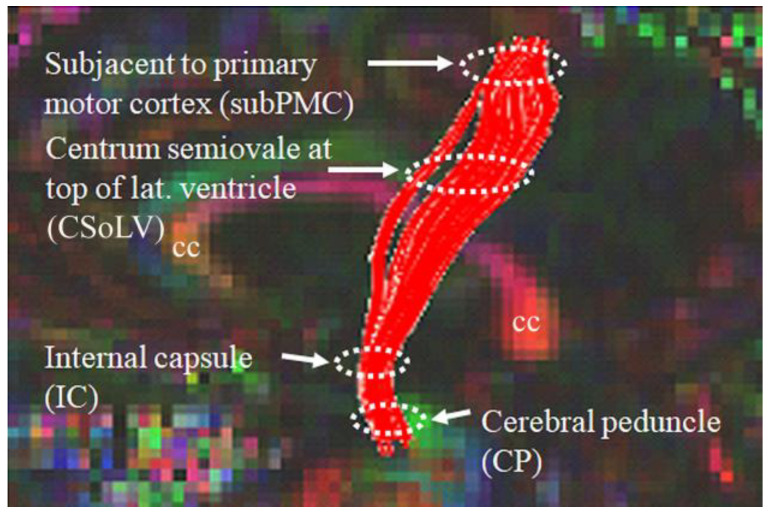
Locations of ROIs along the rostrocaudal extent of the CST where DTI measures were calculated in both neurologic controls and ALS patient subgroups.

Diffusion-weighted images were first corrected for susceptibility artifacts and eddy-current distortion using FSL tools with b-matrix rotation after oblique-angle correction to preserve correct orientation information [20]. These images were then processed using DTI Studio open software in the following manner: (a) diffusion tensor fitting using a multivariate linear least square fit; (b) FA, MD, AD and RD maps of whole brain obtained [20]; (c) fiber tracking using the fiber assignment by continuous tracking (FACT) algorithm; (d) reconstruction of bilateral CST virtual fibers after placing a region of interest (ROI) caudally in the cerebral peduncle (CP) and another ROI rostrally just beneath the primary motor cortex (subPMC); (e) identification of four ROIs at specific CST levels: CP, posterior limb of internal capsule (PLIC), centrum semiovale at top of lateral ventricle (CSoLV), and subPMC, as shown in Figure 2. The aforementioned DTI measures in each ROI bilaterally were measured in ALS patients and neurological controls, as described previously [20]. DTI-derived WM measures along the CST are shown in Table 1, lines 60–91.

### 2.5. Fractal Dimension WM and GM Features

FD analysis was performed using customized in-house routines, as detailed previously [18], and included: (a) extraction of brain precedes its segmention into WM and GM probability maps using FSL tools; (b) binarization of WM and GM probability maps using a threshold value of 0.5; (c) application of 3D thinning to WM and GM binary images to produce corresponding 3D skeleton images [18]; generation of skeleton and general structure of left and right hemispheres after applying hemisphere-specific masks; (d) estimation of FD values using 3D box-counting method. 

FD values of skeleton, surface, and general structure shape representations were estimated by counting boxes required to cover: (a) skeleton foreground voxels for skeleton FD value; (b) WM and GM interface boundary for surface FD; and (c) all WM and GM foreground voxels (which included skeleton and surface) for general structure FD. The skeleton, which preserves topological and geometric information of WM and GM, represents interior structure complexity of brain WM and GM. The surface structure reflects the shape of gyral and sulcal convolutions at the WM–GM interface. The general structure represents volume changes. Because the skeleton, surface and general structure represent three different aspects of brain WM and GM integrity, they may provide insights into brain shape and structural changes occurring during the neurodegeneration of ALS. T1-w imaging-derived FD-based WM and GM measures are shown in Table 1, lines 92–109.

### 2.6. Demographic and Clinical Features

Demographic features of age and gender, as well as clinical characteristics in ALS subgroups of ALSFRS-R, disease duration, El Escorial score, and disease progression rate are shown in Table 1, lines 112–121.

### 2.7. Machine Learning Methods

Based on the article by Grollemund and colleagues [15] that discussed the pros and cons of different ML algorithms employed in ALS, we chose to apply Random Forest (RF) and neural networks (NN) methods. We wrote custom ML codes in MATLAB (Mathworks https://www.mathworks.com/ access date 1 October 2019) version 2016 and also in Python (https://www.python.org/downloads/release/python-390/ access date 3 November 2022) version 3.9 for classification of our ALS phenotypes and controls. Briefly, RF is an ensemble of decision trees, of which a sample of records/data is chosen for each. Gini impurity values were then used to select the best from the attributes and samples for expansion at every node in the decision tree. “Out of bag error” (OOB), which should be as low as possible for accurate prediction, was calculated for each decision tree in RF. Because CST truncation of virtual tracts occurred from DTI calculations in some UMN-predominant ALS patients [5], we filled in the missing data points with the average value of the dataset. Further, we performed feature selection, as suggested in [15], using Waikato Environment for Knowledge (WEKA), which is an ML software developed at the University of Waikato, New Zealand (https://www.cs.waikato.ac.nz/ml/weka/ access date 23 October 2019). Feature selection in WEKA is divided into two parts: attribute evaluator and search method. The feature selection method, CfsSubsetEval, evaluates worthiness of a subset of attributes depending on their individual output predictive power and the level of redundancy between them. We then used the BestFitsearch option to obtain attributes. 

When first employing the classification and regression tree (CART) and pruning algorithms to a data sample size of 106 and 121 attributes, accuracy of classification was only 55%; however, after applying the RF algorithm to this sample, accuracy increased to 71%. For this reason, we used the RF algorithm to analyze our data. Attributes selected by WEKA were then classified by considering each as separate category or output class variables: (1) neurologic controls, ALS-CST+ and ALS-CST− subgroups; and (2) neurologic controls, ALS-Cl and ALS-FTD subgroups.

### 2.8. Neural Networks

We employed neural networks for classification after a custom code was written using MATLAB (Mathworks https://www.mathworks.com/ access date 1 October 2019) version 2016. Briefly, a neural network is a computing model comprising of neurons in different layers. After a literature review, we elected to use a two-layered architecture. The number of neurons in each of the two hidden layers was decided on a case-by-case basis (i.e., when WM or GM attributes were considered separately, clinical and demographic measures were considered, or all of these measures were taken together) using the geometric pyramidal rule proposed by Masters [25] calculations given below:

If we define $r={\left(\frac{\mathrm{Number}\;\mathrm{of}\;\mathrm{input}\;\mathrm{data}}{\mathrm{Number}\;\mathrm{of}\;\mathrm{output}\;\mathrm{data}}\right)}^{1/3}$, then the number of neurons in the

- first hidden layer $=\left(\mathrm{Number}\;\mathrm{of}\;\mathrm{output}\;\mathrm{data}\right)\times r^{2}$
- second hidden layer $=\left(\mathrm{Number}\;\mathrm{of}\;\mathrm{output}\;\mathrm{data}\right)\times r$

Gradient descent with momentum was used for parameter characterization. The value of momentum was fixed at 0.9, training time at 5000, and number of folds for cross-validation was 5.

## 3. Results

### 3.1. Random-Forest-Based Classification

**Figure 3:**
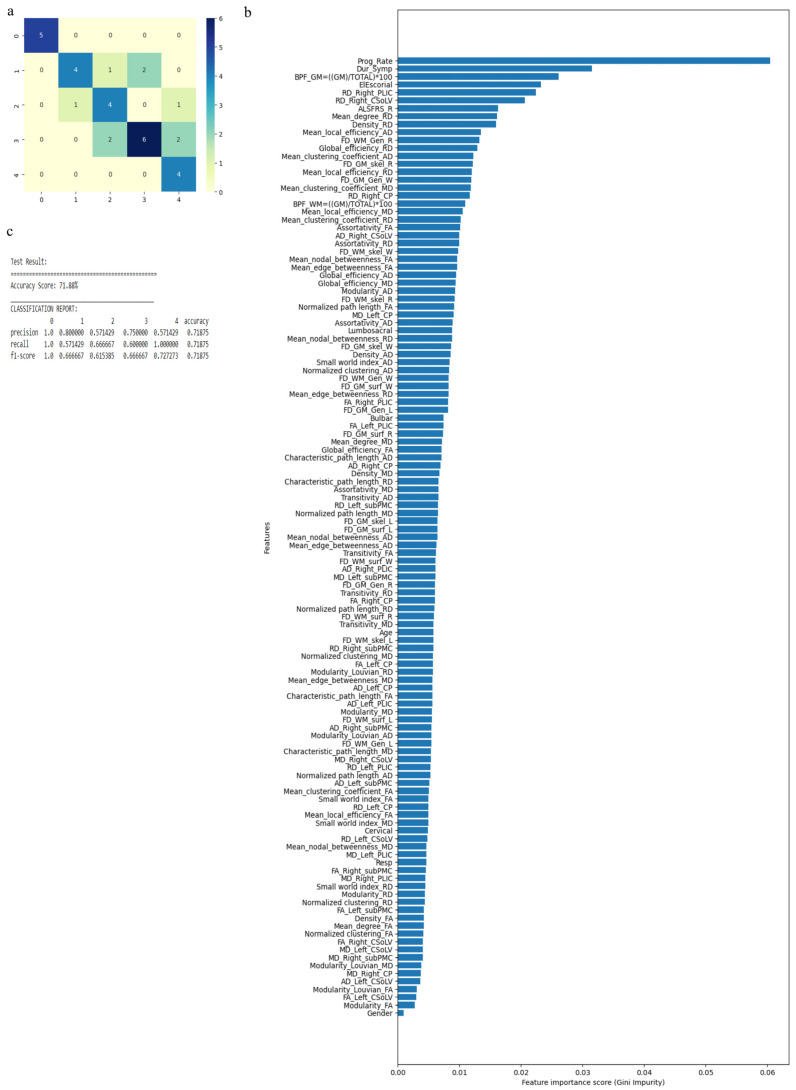
Classification of neurologic controls and ALS subgroups when all 121 features are used, showing the (**a**) confusion matrix, (**b**) variable of importance, and (**c**) statistical measures of precision, recall, and F1 scores. The numbers (0 to 4) shown along the horizontal and vertical axes in (**a**) and labeling the columns of the classification report in (**c**) denote the following subgroups: 0 for neurologic controls, 1 for ALS-CST+, 2 for ALS-CST−, 3 for ALS-Cl, and 4 for ALS-FTD. Abbreviations of feature names used in figure (**b**) include: AD—Axial diffusivity, ALS-FRS_R—Revised ALS function rating scale, BPF—Brain parenchymal fraction, Bulbar—Bulbar subscore (ALSFRS-R), Cervical—Cervical subscore (ALSFRS-R), CP—Cerebral peduncle, CSoLV—Centrum semiovale at top of lateral ventricle, Dur_Symp—Duration of symptoms, ElEscorial—El Escorial score, FA—Fractional anisotropy, FD—Fractal dimension, Gen—General structure, GM—Gray matter, L and Left—Left hemisphere, Lumbosacral—Lumbosacral subscore (ALSFRS-R), MD—Mean diffusivity, PLIC—Posterior limb of internal capsule, Prog_Rate—Progression rate of disease, R and Right —Right hemisphere, RD—Radial diffusivity, Resp—Respiratory subscore (ALSFRS-R), skel—Skeleton of the brain, subPMC—Subcortical primary motor cortex, surf—Surface, W—Whole brain, WM—White matter.

**Figure 4:**
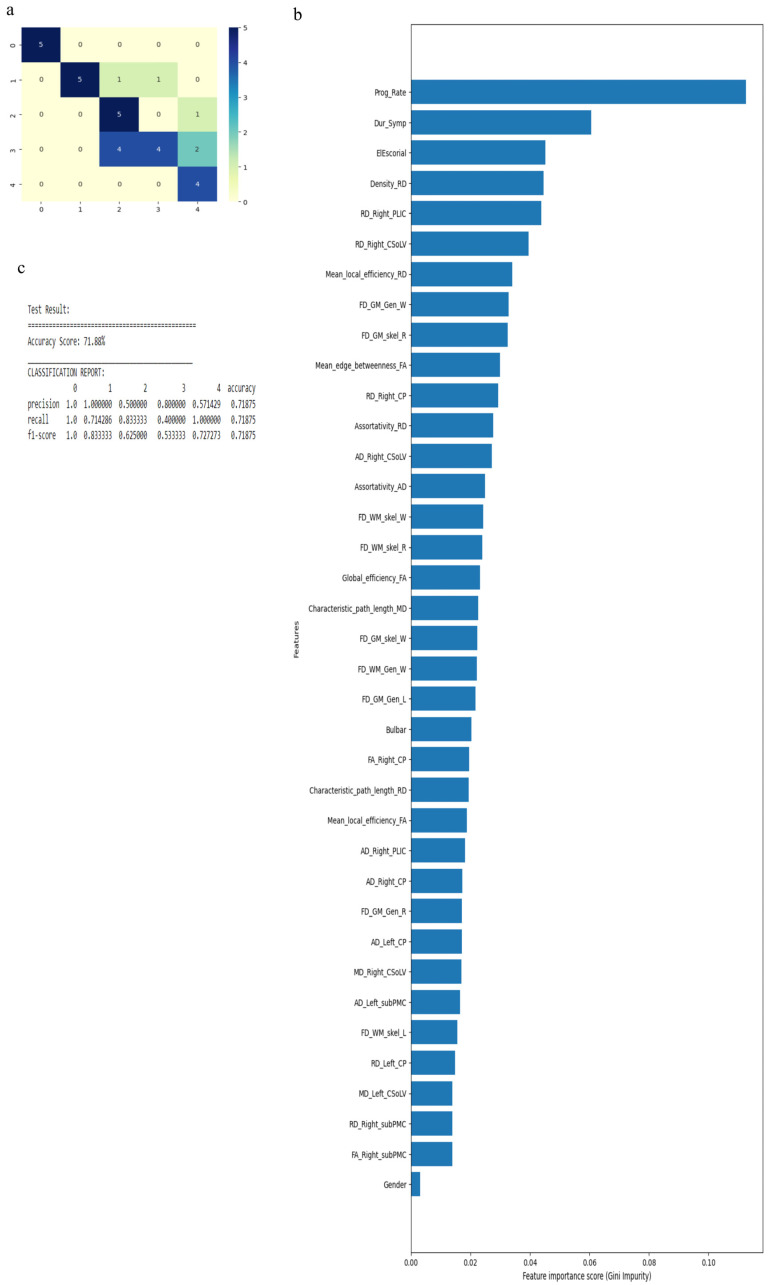
Classification of neurologic controls and ALS subgroups when WEKA-selected attributes are used, showing the (**a**) confusion matrix, (**b**) variable of importance, and (**c**) statistical measures of precision, recall, and F1 scores. The numbers (0 to 4) shown along the horizontal and vertical axes in (**a**) and labeling the columns of the classification report in (**c**) denote the following subgroups: 0 for neurologic controls, 1 for ALS-CST+, 2 for ALS-CST−, 3 for ALS-Cl, and 4 for ALS-FTD. Abbreviations of feature names used in figure (**b**) include: AD—Axial diffusivity, Bulbar—Bulbar subscore (ALSFRS-R), CP—Cerebral peduncle, CSoLV—Centrum semiovale at top of lateral ven-tricle, Dur_Symp—Duration of symptoms, ElEscorial—El Escorial score, FA—Fractional anisotropy, FD—Fractal dimension, Gen—General structure, GM—Gray matter, L and Left—Left hemisphere, MD—Mean diffusivity, PLIC—Posterior limb of internal capsule, Prog_Rate—Progression rate of disease, R and Right—Right hemisphere, RD—Radial diffusivity, skel—Skeleton of the brain, subPMC—Subcortical primary motor cortex, W—Whole brain, WM—White matter.

**Figure 5:**
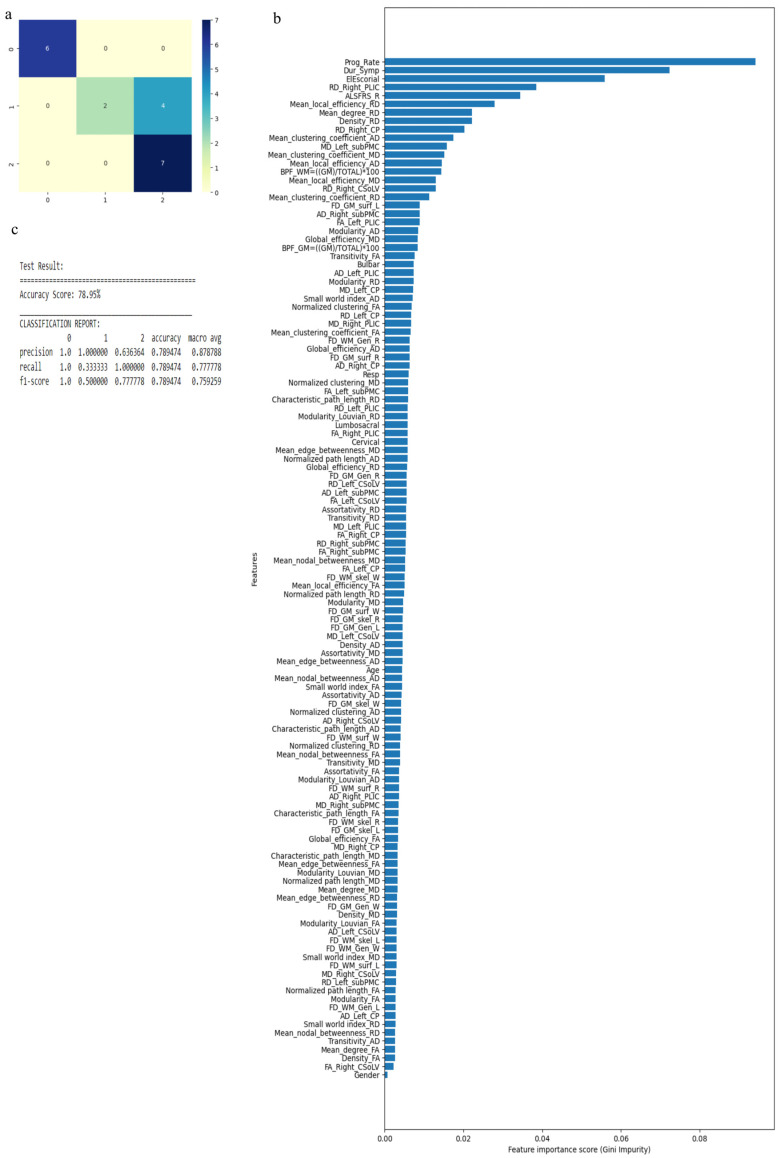
Classification of neurologic controls, ALS-CST+, and ALS-CST− subgroups when all 121 features are used, showing the (**a**) confusion matrix (**b**) variable of importance, and (**c**) statistical measures of precision, recall, and F1 scores. The numbers (0 to 2) shown along the horizontal and vertical axes in (**a**) and labeling the columns of the classification report in (**c**) denote the following subgroups: 0 for neurologic controls, 1 for ALS-CST+, and 2 for ALS-CST−. Abbreviations of feature names used in figure (**b**) include: AD—Axial diffusivity, ALS-FRS_R—Revised ALS function rating scale, BPF—Brain parenchymal fraction, Bulbar—Bulbar subscore (ALSFRS-R), Cervical—Cervical subscore (ALSFRS-R), CP—Cerebral peduncle, CSoLV—Centrum semiovale at top of lateral ventricle, Dur_Symp—Duration of symptoms, ElEscorial—El Escorial score, FA—Fractional anisotropy, FD—Fractal dimension, Gen—General structure, GM—Gray matter, L and Left—Left hemisphere, Lumbosacral—Lumbosacral sub-score (ALSFRS-R), MD—Mean diffusivity, PLIC—Posterior limb of internal capsule, Prog_Rate—Progression rate of disease, R and Right —Right hemisphere, RD—Radial diffusivity, Resp—Respiratory subscore (ALSFRS-R), skel—Skeleton of the brain, subPMC—Subcortical primary motor cortex, surf—Surface, W—Whole brain, WM—White matter.

**Figure 6:**
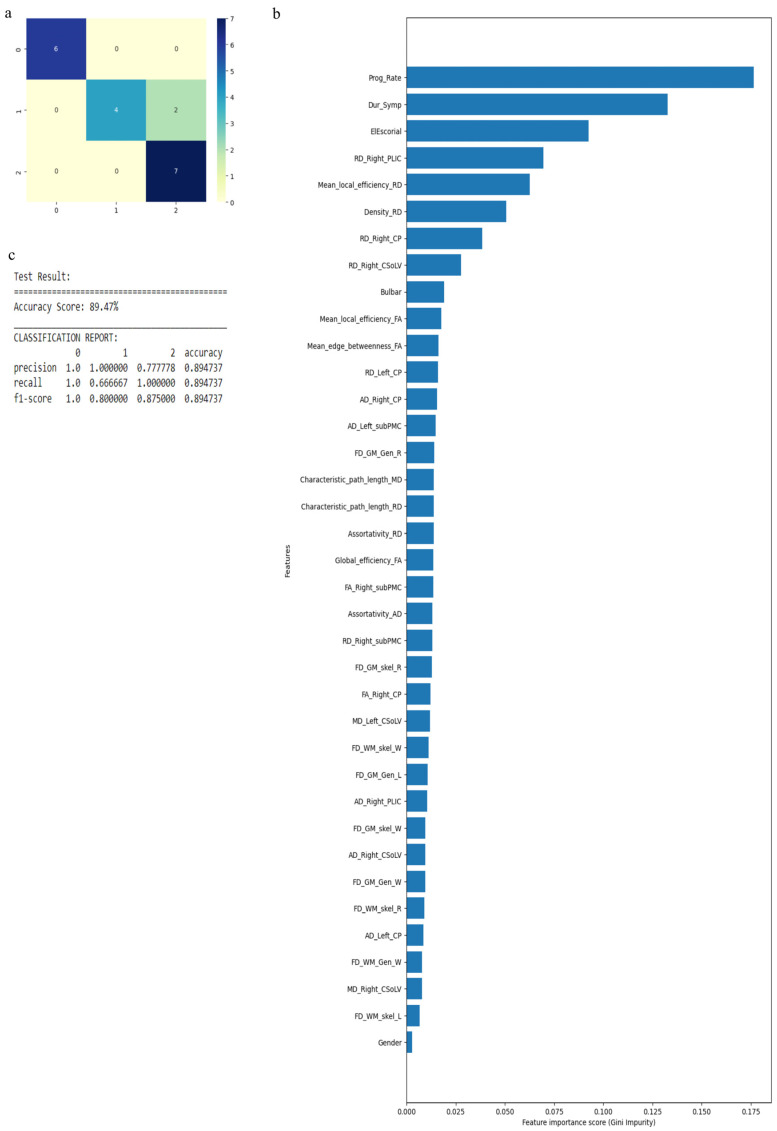
Classification of neurologic controls and ALS subgroups when WEKA-selected attributes are used, showing the (**a**) confusion matrix, (**b**) variable of importance, and (**c**) statistical measures of precision, recall, and F1 scores. The numbers (0 to 4) shown along the horizontal and vertical axes in (**a**) and labeling the columns of the classification report in (**c**) denote the following subgroups: 0 for neurologic controls, 1 for ALS-CST+, and 2 for ALS-CST−. Abbreviations of feature names used in figure (**b**) include: AD—Axial diffusivity, Bulbar—Bulbar subscore (ALSFRS-R), CP—Cerebral peduncle, CSoLV—Centrum semiovale at top of lateral ventricle, Dur_Symp—Duration of symptoms, ElEscorial—El Escorial score, FA—Fractional anisotropy, FD—Fractal dimension, Gen—General structure, GM—Gray matter, L and Left—Left hemisphere, MD—Mean diffusivity, PLIC—Posterior limb of internal capsule, Prog_Rate—Progression rate of disease, R and Right—Right hemisphere, RD—Radial diffusivity, skel—Skeleton of the brain, subPMC—Subcortical primary motor cortex, W—Whole brain, WM—White matter.

The RF algorithm was used to perform the above classification between ALS clinical phenotypes, and average values were used to fill the missing data values. All 121 attributes were considered for this classification and output classes were 0 for control group, 1 for ALS-CST+ subgroup, 2 for ALS-CST− subgroup, 3 for ALS-Cl subgroup, and 4 for ALS-FTD subgroup. Accuracy with RF was 71% and RF classification after attribute selection using the attributes given by WEKA are shown in Table 2. RF classified the ALS clinical phenotypes from controls as well as between the ALS subgroups with 71% accuracy. Accuracy remained at 71% with the reduced attributes when compared to classifying the ALS phenotypes using all 121 attributes. The confusion matrix, statistical parameters and variable of importance when considering all the attributes, and the WEKA-selected attributes are shown in Figure 3 and Figure 4. A comparison between the statistical measures in Figure 3 and Figure 4 shows that attribute selection using WEKA shows some good improvement in the ALS-CST+ and ALS-CST− groups, suggesting that selected features may be better when compared to considering all 121 features for classification. Accuracy increased to 89.5%, however, when the second category for classification comprised of 0 for controls, 1 for ALS CST+, and 2 for ALS CST−, when the attributes given by WEKA (Table 3) were used and average values to fill the missing data points were considered. Accuracy dropped to 78.9% when all 121 attributes were used. The confusion matrix, statistical parameters and variable of importance when considering all the attributes, and the WEKA-selected attributes are shown in Figure 5 and Figure 6. Since the classification of ALS-CST+ and ALS-CST− groups was based on MRI, in order to understand the role played by imaging attributes in classifying the 0, 1, and 2 groups, we performed an analysis where we considered the imaging measures independently from clinical measures. GM measures, when considered independently, resulted in an accuracy of 36%, whereas WM measures alone yielded 73% accuracy, and 76% accuracy was obtained when clinical and demographic measures were considered alone. As mentioned above, when all the measures were combined, the accuracy of the classification between controls, ALS-CST+, and ALS-CST− groups was 88%.

**Figure 7:**
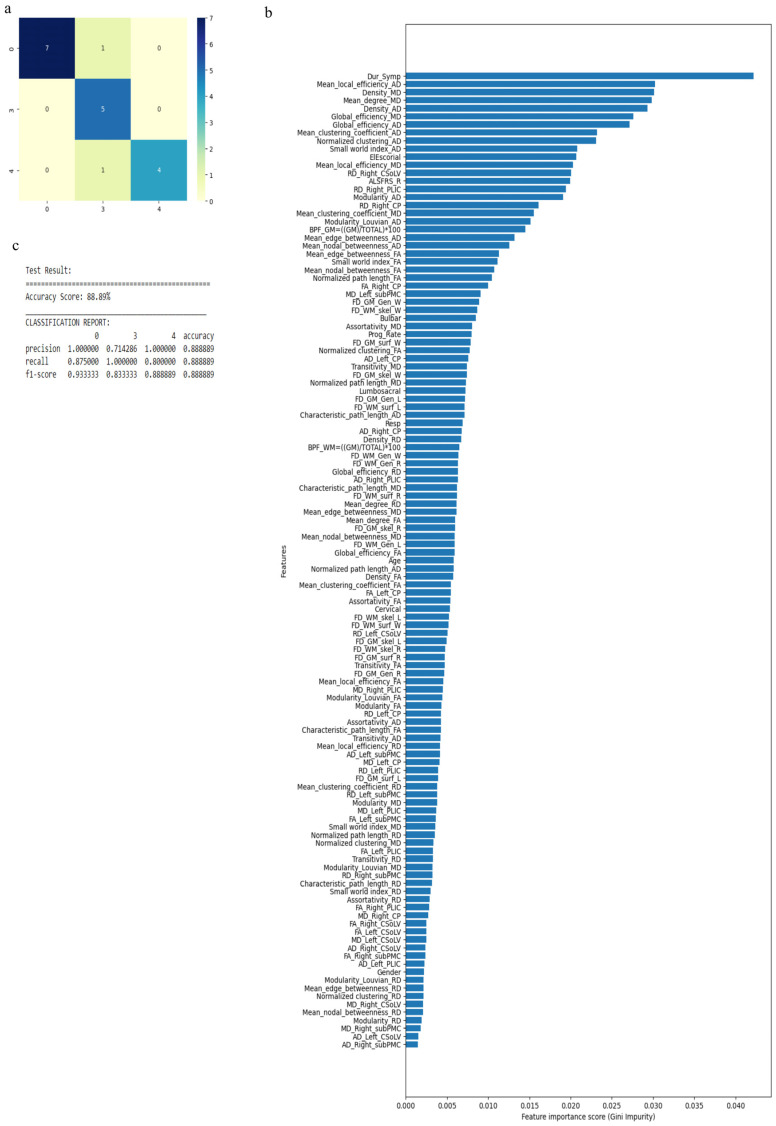
Classification of neurologic controls and ALS subgroups when all 121 features are used, showing the (**a**) confusion matrix, (**b**) variable of importance and (**c**) statistical measures of precision, recall, and F1 scores. The numbers (0, 3, 4) shown along the horizontal and vertical axes in (**a**) and labeling the columns of the classification report in (**c**) denote the following subgroups: 0 for neurologic controls, 3 for ALS-Cl, and 4 for ALS-FTD. Abbreviations of feature names used in figure (**b**) include: AD—Axial diffusivity, ALS-FRS-R—Revised ALS function rating scale, BPF—Brain parenchymal fraction, Bulbar—Bulbar subscore (ALSFRS-R), Cervical—Cervical subscore (ALSFRS-R), CP—Cerebral peduncle, CSoLV—Centrum semiovale at top of lateral ventricle, Dur_Symp—Duration of symptoms, ElEscorial—El Escorial score, FA—Fractional anisotropy, FD—Fractal dimension, Gen—General structure, GM—Gray matter, L and Left—Left hemisphere, Lumbosacral—Lumbosacral sub-score (ALSFRS-R), MD—Mean diffusivity, PLIC—Posterior limb of internal capsule, Prog_Rate—Progression rate of disease, R and Right —Right hemisphere, RD—Radial diffusivity, Resp—Respiratory subscore (ALSFRS-R), skel—Skeleton of the brain, subPMC—Subcortical primary motor cortex, surf—Surface, W—Whole brain, WM—White matter.

**Figure 8:**
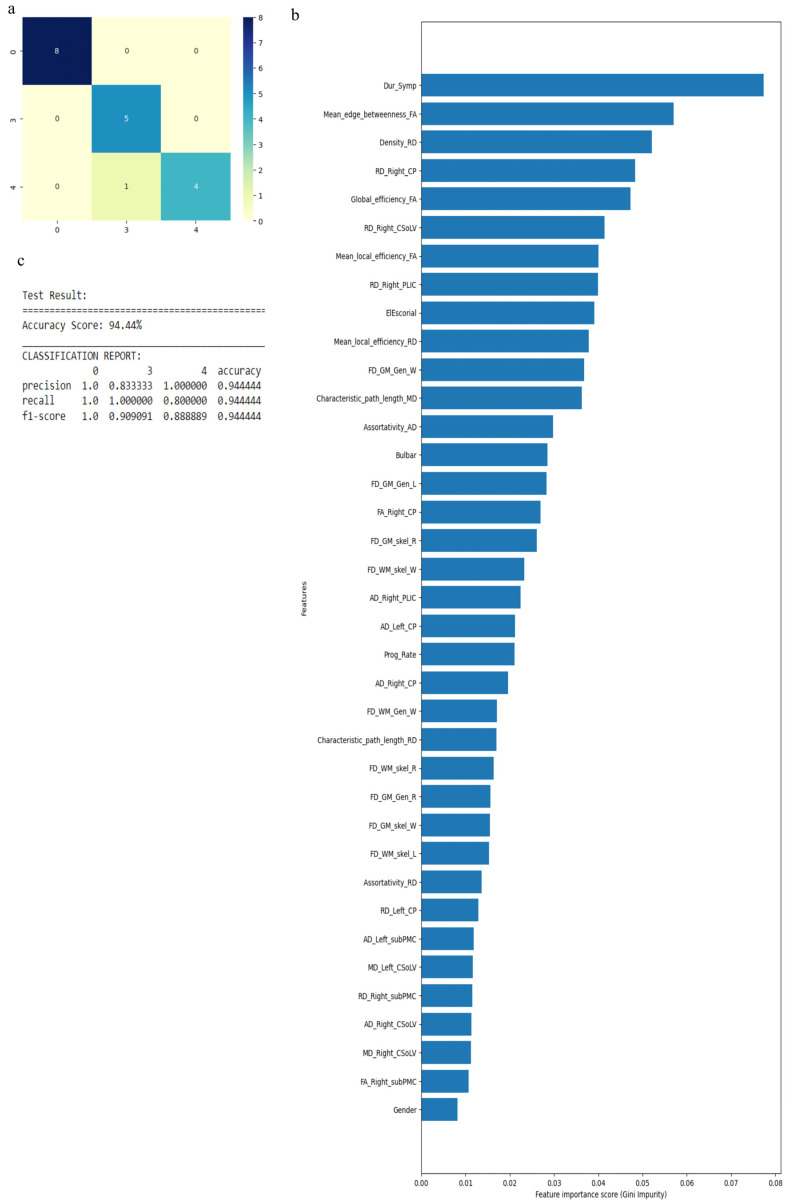
Classification of neurologic controls and ALS subgroups when WEKA-selected attributes are used, showing the (**a**) confusion matrix, (**b**) variable of importance and (**c**) statistical measures of precision, recall, and F1 scores. The numbers (0, 3, 4) shown along the horizontal and vertical axes in (**a**) and labeling the columns of the classification report in (**c**) denote the following subgroups: 0 for neurologic controls, 3 for ALS-Cl, and 4 for ALS-FTD. Abbreviations of feature names used in figure (**b**) include: AD—Axial diffusivity, Bulbar—Bulbar subscore (ALSFRS-R), CP—Cerebral peduncle, CSoLV—Centrum semiovale at top of lateral ventricle, Dur_Symp—Duration of symptoms, ElEscorial—El Escorial score, FA—Fractional anisotropy, FD—Fractal dimension, Gen—General structure, GM—Gray matter, L and Left—Left hemisphere, MD—Mean diffusivity, PLIC—Posterior limb of internal capsule, Prog_Rate—Progression rate of disease, R and Right—Right hemisphere, RD—Radial diffusivity, skel—Skeleton of the brain, subPMC—Subcortical primary motor cortex, W—Whole brain, WM—White matter.

Similarly, when we considered 0 for controls, 3 for ALS-Cl, and 4 for ALS-FTD as well as all 121 attributes with average values to fill the missing data points, accuracy was 88.9%. Accuracy improved to 94.44% when attributes (given by WEKA in Table 4) were used. The confusion matrix, statistical parameters and variable of importance when considering all the attributes, and the WEKA-selected attributes are shown in Figure 7 and Figure 8. We then considered clinical, GM, and WM measures independently. When clinical measures were used to classify all ALS clinical phenotypes, an accuracy of 64% was obtained. The classification of ALS CST+ and ALS CST− patients resulted in accuracy of 75%, and ALS-Cl and ALS-FTD patients resulted in accuracy of 78%. When GM attributes were used to classify the ALS clinical phenotypes and controls, classification accuracy remained very poor (<48%). On the other hand, using WM measures resulted in an accuracy of 73% when comparing between controls, ALS CST+, and ALS CST− patients, and 75% when comparing between controls, ALS-Cl, and ALS-FTD patients; when all ALS clinical phenotypes combined and controls were considered, accuracy dropped to 58%.

### 3.2. Neural-Network-Based Classification

NN-based classification resulted in a low accuracy of 61% when all 121 attributes were used to stratify the 4 ALS patient phenotypes and control group. Selection of features shown in Table 2 slightly improved classification accuracy to 71%. Classifying the second category of the controls, ALS CST+, and ALS CST− subgroups by incorporating all 121 attributes resulted in 75% accuracy; after selecting features shown in Table 3, this increased to 83%. Similarly, when the controls, ALS-Cl, and ALS-FTD subgroups were classified incorporating all 121 attributes, accuracy was 75% but also increased to 83% after using the selected features shown in Table 4. Considering clinical, GM, and WM features independently, the following accuracies were observed: (1) clinical measures alone provided an accuracy of 55% when considering all ALS phenotypes, 88% when classifying ALS-CST+ from ALS-CST− subgroups, and 77% when classifying ALS-Cl from ALS-FTD subgroups; (2) GM measures alone gave an accuracy of only 28% when stratifying controls and all ALS phenotypes; 57% when classifying controls, ALS-CST+, and ALS-CST− subgroups; and 50% when classifying controls, ALS-Cl, and ALS-FTD subgroups; and (3) WM measures alone produced an accuracy of 66% when classifying between controls and all ALS phenotypes; 58% when classifying controls, ALS-CST+, and ALS-CST subgroups; and also 58% when classifying controls, ALS-Cl, and ALS-FTD subgroups.

## 4. Discussion

In this study, we demonstrated the: (1) dominant role of WM metrics in classifying the clinical phenotypes of ALS-CST+ and ALS-CST− from controls as well as from each other; (2) dominant role of clinical, GM, and WM attributes in classifying controls, ALS-Cl, and ALS-FTD clinical phenotypes; (3) use of all 121 attributes (clinical, GM, and WM) to classify controls and all ALS phenotypes resulted in 71% accuracy for both RF and NN methods; and (4) dominant role of AD and RD measures in classifying ALS phenotypes, and suggesting that axonal and myelin damage, respectively, occur differentially in ALS.

In the classification of all ALS phenotypes and controls, assortativity, global and local efficiency, mean edge betweenness, normalized path length, and density played important roles in patient stratification among different graph metrics. The assortativity metric reflects the tendency of nodes to connect with other nodes of similar properties (e.g., high-degree nodes connect with other high-degree nodes), whereas negative assortativity (or disassortativity) indicates nodes of dissimilar properties connecting (e.g., high-degree nodes connect with lower-degree nodes). Mean assortativity values of controls and ALS subgroups for AD and RD graph networks, as shown in Supplementary Table S1, revealed differing machine-learning-selected attributes between patient subgroups. Specifically, values among ALS-CST+, ALS-CST−, and ALS-Cl subgroups showed positive assortativity but slightly reduced values when compared to controls, which may reflect compensatory mechanisms by the disrupted brain network. AD-weighted graph-network disassortativity in ALS-FTD patients may be compensatory for functional deficits occurring during loss of WM connections in the frontal and temporal lobes [4]. In a longitudinal ALS study using functional magnetoencephalography, Sorrentino et al. [26] observed high disassortativity with disease progression, which suggested that more peripheral nodes connect to higher-degree nodes to compensate for dysfunctional degenerating brain areas.

Differences in certain DTI-metric attributes at various CST-rostrocaudal levels (see Figure 2) were important in classifying ALS-CST+, ALS-CST−, ALS-FTD, and ALS-Cl subgroups, including RD and MD at CP, CSoLV, and subPMC levels, and AD at the CSoLV level. The ability of these metrics to differentiate the ALS phenotypes infers that distinct patterns of CST degeneration occur in these patient subgroups. In addition, dominance of right hemisphere FA, AD, RD, and MD values in classifying the disease process suggests that CST fibers are affected differentially between hemispheres, more so on the right.

Graph theory measures involved in classifying controls, ALS-CST+, and ALS-CST− groups (as shown in Table 3) included assortativity_AD, transitivity_FA, characteristic path length_FA, mean degree_MD, normalized clustering coefficient_MD, and normalized path length_RD. Transitivity and clustering coefficients reflect local structural (topology-wise) segregation of nodes for functional integration, whereas path length reflects global integration of information in a network. Mean value differences of the above measures were greater between controls and ALS-CST− patients than between controls and ALS-CST+ patients, as seen in Supplementary Table S2. In the ALS-CST− subgroup, increases of characteristic and normalized path length values suggests compromised global integration, whereas decreased assortativity values imply greater interconnection between low-degree and high-degree nodes.

Interestingly, FA, AD, RD, and MD values at only rostral levels of the CST (in CSoLV and subPMC) were used by the feature selection algorithm to classify controls, ALS-CST−, and ALS-CST+ subgroups, while those at caudal levels (in PLIC and CP) were not. Fractal dimension (FD) attributes of WM or GM were not involved in classification. The DTI metrics along the CST revealed abnormal values in both ALS-CST+ and ALS-CST− subgroups when compared to controls and between these two patient subgroups. Mean values of the chosen DTI attributes for control group and UMN-predominant ALS subgroups are shown in Supplementary Table S3. The observation that essentially all DTI metrics along the CST chosen for classification by the feature selection algorithm were from the right hemisphere (except for MD in the left CP) emphasizes the asymmetry of hemispheric involvement, as mentioned above. In addition, lack of GM attributes being chosen for classification and no significant difference of GM FD values between controls, ALS-CST+, and ALS-CST− subgroups suggests either a primary ‘axonopathy’ (and not a ‘neuronopathy’) or insensitivity of GM measures in these UMN-predominant ALS patients. Clinical attributes that played a key role in classifying ALS-CST+ and ALS-CST− subgroups include symptom duration, El Escorial criteria score, and ALSFRS-R subscores of bulbar and lumbosacral function; mean values of these measures are shown in Supplementary Table S4.

Graph theory measures used to classify controls, ALS-Cl, and ALS-FTD subgroups (as shown in Table 4), resulted in accuracies of 88% for RF and 83% for NN. Except for attribute #7, these were all different from those used to classify controls, ALS-CST+, and ALS-CST− subgroups (discussed above), and included: (1) density of the AD network (density_AD), (2) mean local efficiency (mean local efficiency_AD), (3) mean edge betweenness (mean edge betweenness_AD), (4) density based on MD network (density_MD), (5) mean clustering coefficient (mean clustering coefficient_MD), (6) mean local efficiency (mean local efficiency_MD), and (7) normalized path length of the RD network (normalized path length_RD). Network density measures the proportion of connections relative to the total number of possible connections. Mean value differences of the above measures were lower in ALS-FTD patients than in controls and ALS-Cl patients, as shown in Supplementary Table S5. Edge betweenness, local efficiency, and density metrics were important in classifying ALS-FTD from ALS-Cl and controls. Globally lower graph theory metric values in ALS-FTD patients compared to controls and ALS-Cl patients indicate more prominent WM abnormalities in the former patient subgroup. Attributes from DTI measures along the CST involved in classifying ALS-Cl and ALS-FTD subgroups, and controls were not restricted to its caudal level (as when classifying ALS-CST+ and ALS-CST− subgroups) but involved its entire rostrocaudal extent, as shown in Supplementary Table S6. FD WM and GM metrics were important in classifying ALS-Cl and ALS-FTD patients and controls with their mean values, as shown in Supplementary Table S7. Interestingly, WM fractal values were reduced in ALS-FTD patients compared to ALS-Cl patients and controls, whereas GM fractal values were increased in ALS-Cl and ALS-FTD patients when compared to controls. Increased FD GM values suggest an amorphous change in GM structures, as could occur with loss of neurons and their processes. Importance of GM features in classifying ALS-Cl and ALS-FTD patients and controls suggests that neurodegeneration in ALS-FTD (and possibly ALS-Cl) patients may be primarily a ‘neuronopathy’. The mean values of clinical attributes that played a key role in classifying ALS-Cl and ALS-FTD subgroups including symptom duration, El Escorial score, and ALSFRS-R are shown in Supplementary Table S8.

## 5. Conclusions

The RF algorithm was the most accurate in classifying the four different ALS phenotypes. WM metrics played a major role in classifying these phenotypes compared to GM or clinical measures. Although WM measures from both right and left hemispheres should be considered when identifying ALS phenotypes (consistent with bihemispheric degeneration occurring in ALS), they appear to be differentially affected by the disease process. Longitudinal studies of multiple time-point data can confirm and expand on our findings.

Increasing patient numbers in each subgroup, potentially through a multisite study will strengthen the statistical validity of our present findings. Time constraints in obtaining MRI under a clinical protocol prevented the use of smaller voxel sizes and higher-direction-number DTI acquisitions. Follow-up studies using higher-resolution imaging parameters at 3T will also be useful to confirm our findings. As a biomarker, neuroimaging is useful not only in cross-sectional studies (as ours here) for diagnosis and identification of abnormalities present at a single timepoint, but also in multiple timepoint analyses. Therefore, future longitudinal studies of various patient subgroups beginning even earlier in the course of ALS will provide insights into the evolution and spread of disease throughout the brain.

## Figures and Tables

**Table 1:** Total of 121 features and attributes considered in this study for all ALS subgroups and controls.

No.	Measures/Attributes	Type of Attribute (WM/GM/Demographic/Clinical)
1	Assortativity_AD	WM measures from graph theory networks using DTI
2	Density_AD	
3	Mean_clustering_coefficient_AD	
4	Transitivity_AD	
5	Global_efficiency_AD	
6	Mean_local_efficiency_AD	
7	Modularity_AD	
8	Modularity_Louvian_AD	
9	Characteristic_path_length_AD	
10	Mean_nodal_betweenness_AD	
11	Mean_edge_betweenness_AD	
12	Normalized path length_AD	
13	Normalized clustering_AD	
14	Small world index_AD	
15	Mean_degree_FA	
16	Assortativity_FA	
17	Density_FA	
18	Mean_clustering_coefficient_FA	
19	Transitivity _FA	
20	Global_efficiency_FA	
21	Mean_local_efficiency_FA	
22	Modularity_FA	
23	Modularity_Louvian_FA	
24	Characteristic_path_length _FA	
25	Mean_nodal_betweenness_FA	
26	Mean_edge_betweenness_FA	
27	Normalized path length_FA	
28	Normalized clustering_FA	
29	Small world index_FA	
30	Mean_degree_MD	
31	Assortativity_MD	
32	Density_MD	
33	Mean_clustering_coefficient_MD	
34	Transitivity_MD	
35	Global_efficiency_MD	
36	Mean_local_efficiency_MD	
37	Modularity_MD	
38	Modularity_Louvian_MD	
39	Characteristic_path_length_MD	
40	Mean_nodal_betweenness_MD	
41	Mean_edge_betweenness_MD	
42	Normalized path length_MD	
43	Normalized clustering_MD	
44	Small world index_MD	
45	Mean_degree_RD	
46	Assortativity_RD	
47	Density_RD	
48	Mean_clustering_coefficient_RD	
49	Transitivity_RD	
50	Global_efficiency_RD	
51	Mean_local_efficiency_RD	
52	Modularity_RD	
53	Modularity_Louvian_RD	
54	Characteristic_path_length _RD	
55	Mean_nodal_betweenness_RD	
56	Mean_edge_betweenness_RD	
57	Normalized path length_RD	
58	Normalized clustering_RD	
59	Small world index_RD	
60	FA_Right_CP	WM measures along CST using DTI
61	FA_Right_PLIC	
62	FA_Right_ CSoLV	
63	FA_Right_subPMC	
64	FA_Left_CP	
65	FA_Left_PLIC	
66	FA_Left_CSoLV	
67	FA_Left_subPMC	
68	AD_Right_CP	
69	AD_Right_PLIC	
70	AD_Right_CSoLV	
71	AD_Right_subPMC	
72	AD_Left_CP	
73	AD_Left_PLIC	
74	AD_Left_CSoLV	
75	AD_Left_subPMC	
76	RD_Right_CP	
77	RD_Right_PLIC	
78	RD_Right_CSoLV	
79	RD_Right_subPMC	
80	RD_Left_CP	
81	RD_Left_PLIC	
82	RD_Left_CSoLV	
83	RD_Left_subPMC	
84	MD_Right_CP	
85	MD_Right_PLIC	
86	MD_Right_CSoLV	
87	MD_Right_subPMC	
88	MD_Left_CP	
89	MD_Left_PLIC	
90	MD_Left_CSoLV	
91	MD_Left_subPMC	
92	FD_WM_Gen_L	FD-based WM measures from T1-w imaging
93	FD_WM_Gen_R	
94	FD_WM_Gen_W	
95	FD_WM_surf_L	
96	FD_WM_surf_R	
97	FD_WM_surf_W	
98	FD_WM_skel_L	
99	FD_WM_skel_R	
100	FD_WM_skel_W	
101	FD_GM_Gen_L	FD-based GM measures from T1-w imaging
102	FD_GM_Gen_R	
103	FD_GM_Gen_W	
104	FD_GM_surf_L	
105	FD_GM_surf_R	
106	FD_GM_surf_W	
107	FD_GM_skel_L	
108	FD_GM_skel_R	
109	FD_GM_skel_W	
110	BPF_WM = ([WM]/TOTAL) ∗ 100	BPF measures from T1-w imaging
111	BPF_GM = ([GM]/TOTAL) ∗ 100	
112	Age	Demographics
113	Gender	
114	El_Escorial	Clinical measures of ALS
115	Dur_Symp	
116	ALSFRS-R	
117	Bulbar	
118	Cervical	
119	Lumbosacral	
120	Resp	
121	Prog_Rate	

ALSFRS-R—Revised ALS functional rating scale, AD—Axial diffusivity, BPF—Brain parenchymal fraction, Bulbar—Bulbar subscore (ALSFRS-R), Cervical—Cervical subscore (ALSFRS-R), CP—Cerebral peduncle, CSoLV—Centrum semiovale at top of lateral ventricle , DTI—Diffusion tensor imaging, Dur_Symp—Duration of symptoms, El_Escorial—El Escorial score, FD—Fractal dimension, FA—Fractional anisotropy, Gen—General structure, GM—Gray matter, L and Left—Left hemisphere, Lumbosacral—Lumbosacral subscore (ALSFRS-R), MD—Mean diffusivity, PLIC—Posterior limb of the internal capsule, Prog_Rate—Progression rate of disease, R and Right —Right hemisphere, RD—Radial diffusivity, Resp—Respiratory subscore (ALSFRS-R), skel—Skeleton of the brain, subPMC—Subcortical primary motor cortex, Surf—Brain surface, T1-w—T1-weighted, W—Whole brain, WM—White matter.

**Table 2:** Features and attributes selected using WEKA when considering all the ALS subgroups and controls.

No.	Measures/Attributes	Type of Attribute (WM/GM/Demographic/Clinical)
1	Assortativity_AD	WM measures from graph theory networks using DTI
2	Global_effiiciency_FA	
3	Mean_local_efficiency_FA	
4	Mean_edge_betweenness_FA	
5	Normalized path length_MD	
6	Assortativity_RD	
7	Density_RD	
8	Mean_local_efficiency_RD	
9	Normalized path length_RD	
10	FA_Right_CP	WM measures along CST using DTI
11	FA_Right_subPMC	
12	AD_Right_CP	
13	AD_Right_PLIC	
14	AD_Right_CSoLV	
15	AD_Left_CP	
16	AD_Left_subPMC	
17	RD_Right_CP	
18	RD_Right_PLIC	
19	RD_Right_CSoLV	
20	RD_Right_subPMC	
21	RD_Left_CP	
22	MD_Right_CSoLV	
23	MD_Left_CSoLV	
24	FD_WM_Gen_W	FD-based GM and WM measures from T1-w imaging
25	FD_WM_skel_L	
26	FD_WM_skel_R	
27	FD_WM_skel_W	
28	FD_GM_Gen_L	
29	FD_GM_Gen_R	
30	FD_GM_Gen_W	
31	FD_GM_skel_R	
32	FD_GM_skel_W	
33	Gender	Demographic and clinical measures
34	El_Escorial	
35	Dur_Symp	
36	Bulbar	
37	Prog_Rate	

AD—Axial diffusivity, BPF—Brain parenchymal fraction, Bulbar—Bulbar subscore (ALSFRS-R), CP—Cerebral peduncle, CSoLV—Centrum semiovale at top of lateral ventricle, DTI—Diffusion tensor imaging, Dur_Symp—Duration of symptoms, El_Escorial—El Escorial score, FD—Fractal dimension, FA—Fractional anisotropy, Gen—General structure, GM—Gray matter, L and Left—Left hemisphere, MD—Mean diffusivity, PLIC—Posterior limb of the internal capsule, Prog_Rate—Disease progression rate, R and Right—Right hemisphere , RD—Radial diffu-sivity, skel—Skeleton of the brain, subPMC—Subcortical primary motor cortex, T1-w—T1-weighted, W—Whole brain, WM—White matter.

**Table 3:** Features and attributes selected using WEKA when considering only ALS-CST+ and ALS-CST− subgroups and controls.

No.	Measures/Attributes	Type of Attribute (WM/Demographic/Clinical)
1	Assortativity_AD	WM measures from graph theory networks using DTI
2	Transitivity_FA	
3	Characteristic_path_length_FA	
4	Mean_degree_MD	
5	Normalized clustering_MD	
6	Normalized path length_RD	
7	FA_Right_CP	
8	AD_Right_CP	WM measures along CST using DTI
9	AD_Right_CSoLV	
10	RD_Right_CP	
11	RD_Right_PLIC	
12	RD_Right_CSoLV	
13	RD_Right_subPMC	
14	MD_Right_CP	
15	MD_Left_CP	
16	Gender	Demographic and clinical measures
17	El_Escorial	
18	Dur_Symp	
19	Bulbar	
20	Lumbosacral	

AD—Axial diffusivity, CP—cerebral peduncle, DTI—Diffusion tensor imaging, Dur_Symp—Duration of symptoms, FA—Fractional anisotropy, Left—Left hemisphere, MD—Mean diffusivity, PLIC—Posterior limb of the internal capsule, RD—Radial diffusivity, Right—Right hemisphere, subPMC—Subcortical primary motor cortex, CSoLV—Centrum semiovale at top of lateral ventricle, WM—White matter.

**Table 4:** Features and attributes selected using WEKA when considering only ALS-FTD and ALS-Cl subgroups and controls.

No.	Measures/Attributes	Type of Attribute (WM/GM/Demographic/Clinical)
1	Density_AD	WM measures from graph theory networks using DTI
2	Mean_local_efficiency_AD	
3	Mean_edge_betweenness_AD	
4	Density_MD	
5	Mean_clustering_coefficient_MD	
6	Mean_local_efficiency_MD	
7	Normalized path length_RD	
8	FA_Left_PLIC	
9	FA_Left_CSoLV	WM measures from CST using DTI
10	FA_Left_subPMC	
11	AD_Right_CP	
12	AD_Right_PLIC	
13	RD_Right_CP	
14	RD_Right_PLIC	
15	RD_Right_CSoLV	
16	RD_Right_subPMC	
17	MD_Right_CP	
18	MD_Left_CP	
19	MD_Left_subPMC	
20	FD_WM_skel_W	FD-based GM and WM measures from T1-w imaging
21	FD_GM_Gen_R	
22	FD_GM_skel_L	
23	FD_GM_skel_R	
24	FD_GM_skel_W	
25	El_Escorial	Demographic and clinical measures
26	Dur_Symp	
27	ALSFRS-R	

ALSFRS-R—Revised ALS functional rating scale, AD—Axial diffusivity, CP—Cerebral peduncle, CSoLV—Centrum semiovale at top of lateral ventricle, DTI—Diffusion tensor imaging, Dur_Symp—Duration of symptoms, El_Escorial—El Escorial score, FD—Fractal dimension, FA—Fractional anisotropy, Gen—General structure, GM—Gray matter, L and Left—Left hemi-sphere, MD—Mean diffusivity, PLIC—Posterior limb of the internal capsule, R and Right—Right hemisphere, RD—Radial diffusivity, skel—Skeleton of the brain, subPMC—Subcortical primary motor cortex, Surf—Brain surface, T1-w—T1-weighted, W—Whole brain, WM—White matter.

## Data Availability

The data used in this study are property of Cleveland Clinic and therefore cannot be shared.

## Supplementary Materials

The following supporting information can be downloaded at: https://www.mdpi.com/article/10.3390/diagnostics13091521/s1, Table S1: Mean assortativity values of the control group and ALS subgroups; Table S2: Mean graph measure values in the control group and UMN-predominant ALS subgroups; Table S3: Mean DTI metrics along the CST in the control group and UMN-predominant ALS subgroups; Table S4: Clinical measures influencing classification of UMN-predominant ALS subgroups; Table S5: Mean graph measure values in controls and patients with classic ALS and ALS-FTD; Table S6: Mean DTI metrics along the CST in controls and patients with classic ALS and ALS-FTD; Table S7: Mean FD attributes influencing classification of controls and patients with classic ALS and ALS-FTD; Table S8: Clinical measures influencing classification of patients with classic ALS and ALS-FTD.
