# Supplementary material for: Quantitative Brain MRI Metrics Distinguish Four Different ALS Phenotypes: A Machine Learning Based Study
Source: Diagnostics (Basel). 2023 Apr 24;13(9):1521. doi: 10.3390/diagnostics13091521 (PMC10177762; doi:10.3390/diagnostics13091521)
Supplement: Supplementary file 1 [file diagnostics-13-01521-s001.zip › diagnostics-2274140-supplementary materials.pdf]

Supplementary Materials

Supplementary Table S1 - Mean assortativity values of the control group and ALS subgroups

**Patient Group/Graph Measure**	**Control**	**ALS-CST+**	**ALS-CST–**	**ALS-Cl**	**ALS-FTD**
Assortativity Value from AD Graph	0.12	0.17	0.04	0.10	–0.04
Assortativity Value from RD Graph	0.07	0.05	0.06	0.05	0.05

Abbreviations used in Supplementary Table S1:

ALS-Cl - Classic ALS patients, ALS-CST+ - UMN-predominant ALS patients with CST hyperintensity, ALS-CST– - UMN-predominant ALS patients without CST hyperintensity, ALS-FTD - ALS patients with frontotemporal dementia, AD - Axial diffusivity, RD - Radial diffusivity.

Supplementary Table S2 – Mean graph measure values in the control group and UMN-predominant ALS subgroups

Patient Group/DTI network metric	Graph Measure	Controls	ALS-CST+	ALS-CST–
FA	Transitivity	0.53	0.53	0.59
	Characteristic path length	2.40	2.53	2.27
MD	Mean degree value	7.83	5.93	3.97
	Normalized clustering coefficient	3.34	2.96	2.51
RD	Normalized path length	1.17	1.14	1.21

Abbreviations used in Supplementary Table S2:

ALS-CST+ - UMN-predominant ALS patients with CST hyperintensity, ALS-CST– - UMN-predominant ALS patients without CST hyperintensity, DTI - Diffusion tensor imaging, FA - Fractional anisotropy, MD - Mean diffusivity, RD - Radial diffusivity.

Supplementary Table S3 - Mean DTI metrics along the CST in the control group and UMN-predominant ALS subgroups

**Patient Group/DTI network metrics**	**ROI location along CST**	**Controls**	**ALS-CST+**	**ALS-CST–**
AD (mm^2/sec)	Right CP	1.64×10^{-3}	1.78×10^{-3}	1.89×10^{-3}
	Right CSoLV	1.10×10^{-3}	1.09×10^{-3}	1.16×10^{-3}
RD (mm^2/sec)	Right CP	0.49×10^{-3}	0.76×10^{-3}	0.86×10^{-3}
	Right PLIC	0.36×10^{-3}	0.54×10^{-3}	1.33×10^{-3}
	Right CSoLV	0.5×10^{-3}	0.6×10^{-3}	0.6×10^{-3}
	Right subPMC	0.52×10^{-3}	0.34×10^{-3}	0.64×10^{-3}
MD (mm^2/sec)	Right CP	0.87×10^{-3}	0.88×10^{-3}	0.92×10^{-3}
	Left CP	1.10×10^{-3}	0.90×10^{-3}	0.90×10^{-3}

Abbreviations used in Supplementary Table S3:

ALS-CST+ - UMN-predominant ALS patients with CST hyperintensity, ALS-CST– - UMN-predominant ALS patients without CST hyperintensity, AD - Axial diffusivity, CP - Cerebral peduncle, CST - corticospinal tract, DTI - Diffusion tensor imaging, FA - Fractional anisotropy, MD - Mean diffusivity, PLIC - Posterior limb of internal capsule, RD - Radial diffusivity, ROI - Region of interest, subPMC - Subcortical to primary motor cortex, CSoLV—Centrum semiovale at top of lateral ventricle.

Supplementary Table S4 - Clinical measures influencing classification of UMN-predominant ALS subgroups

**Patient Group/Clinical Measures**	**ALS-CST+**	**ALS-CST–**
Symptom duration (mos)	14.3	59.3
El Escorial criteria	1.37	1.81
Bulbar component of ALSFRS-R score	10.06	9.47
Lumbosacral component of ALSFRS-R score	6.13	6.79

Abbreviations used in Supplementary Table S4:

ALS-CST+ - UMN-predominant ALS patients with CST hyperintensity, ALS-CST– - UMN-predominant ALS patients without CST hyperintensity, ALSFRS-R - Revised ALS functional rating scale, mos - months.

Supplementary Table S5 – Mean graph measure values in controls, and patients with classic ALS and ALS-FTD

Patient Group/DTI network metric	Graph Measure	Controls	ALS-Cl	ALS-FTD
AD	Network density	0.07	0.06	0.03
	Mean local efficiency	0.59	0.55	0.29
	Mean edge betweenness	1.30	1.16	0.50
MD	Network density	0.07	0.06	0.03
	Mean clustering coefficient	0.49	0.46	0.25
	Mean local efficiency	0.60	0.56	0.30

Abbreviations used in Supplementary Table S5:

ALS-Cl - Classic ALS patients, ALS-FTD - ALS patients with frontotemporal dementia, AD - Axial diffusivity, DTI - Diffusion tensor imaging, MD - Mean diffusivity.

Supplementary Table S6 – Mean DTI metrics along the CST in controls, and patients with classic ALS and ALS-FTD

Patient Group/DTI measures along CST	ROI along CST	Controls	ALS-Cl	ALS-FTD
FA	Left PLIC	0.76	0.72	0.70
	Left CSoLV	0.51	0.46	0.46
	Left SubPMC	0.66	0.54	0.56
AD (mm^2/sec)	Right CP	1.60×10^{-3}	1.90×10^{-3}	2.0×10^{-3}
	Right PLIC	1.50×10^{-3}	1.50×10^{-3}	1.70×10^{-3}
RD (mm^2/sec)	Right CP	0.49×10^{-3}	0.80×10^{-3}	0.98×10^{-3}
	Right PLIC	0.36×10^{-3}	0.49×10^{-3}	1.11×10^{-3}
	Right CSoLV	0.50×10^{-3}	0.60×10^{-3}	0.60×10^{-3}
	Right subPMC	0.52×10^{-3}	0.60×10^{-3}	0.68×10^{-3}
MD (mm^2/sec)	Right CP	0.87×10^{-3}	0.86×10^{-3}	0.94×10^{-3}
	Left CP	1.10×10^{-3}	0.95×10^{-3}	0.91×10^{-3}
	Left subPMC	0.90×10^{-3}	0.73×10^{-3}	0.69×10^{-3}

Abbreviations used in Supplementary Table S6:

ALS-Cl - Classic ALS patients, ALS-FTD - ALS patients with frontotemporal dementia, AD - Axial diffusivity, CP - Cerebral peduncle, CST - corticospinal tract, DTI - Diffusion tensor imaging, FA - Fractional anisotropy, MD - Mean diffusivity, PLIC - Posterior limb of internal capsule, RD - Radial diffusivity, ROI - Region of interest, subPMC - Subcortical to primary motor cortex, CSoLV—Centrum semiovale at top of lateral ventricle.

Supplementary Table S7 - Mean FD attributes influencing classification of controls and patients with classic ALS and ALS-FTD

**Patient Group/FD measures**	**Controls**	**ALS-Cl**	**ALS-FTD**
(FD_WM_skel_W)	2.49	2.48	2.47
(FD_GM_Gen_R)	2.56	2.57	2.57
(FD_GM_skel_L)	2.47	2.47	2.48
(FD_GM_skel_R)	2.48	2.48	2.49
(FD_GM_skel_W)	2.55	2.56	2.56

Abbreviations used in Supplementary Table S7:

ALS-Cl - Classic ALS patients, ALS-FTD - ALS patients with frontotemporal dementia, FD - Fractal dimension, Gen - Brain general structure, GM - Gray matter, Left and L - Left hemisphere, R, Right - Right hemisphere, Skel - Skeleton of the brain, WM - White matter, W - Whole brain.

Supplementary Table S8 - Clinical measures influencing classification of patients with classic ALS and ALS-FTD

**Patient Group/Clinical Measures**	**ALS-Cl**	**ALS-FTD**
Symptom duration (mos)	29.1	35.1
El Escorial criteria score	2.48	2.41
ALSFRS-R score	37.2	30.5

Abbreviations used in Supplementary Table S8:

ALS-Cl - Classic ALS patients, ALS-FTD - ALS patients with frontotemporal dementia, ALSFRS-R - Revised ALS functional rating scale, mos - months.
